# Thermoelectric properties of Cu-dispersed bi_0.5_sb_1.5_te_3_

**DOI:** 10.1186/1556-276X-7-2

**Published:** 2012-01-05

**Authors:** Il-Ho Kim, Soon-Mok Choi, Won-Seon Seo, Dong-Ik Cheong

**Affiliations:** 1 Department of Materials Science and Engineering, Chungju National University, Chungju, Chungbuk 380-702, South Korea; 2 Energy Materials Lab., Green Ceramic Division, Korea Institute of Ceramic Engineering and Technology, Seoul 153-801, South Korea; 3 The 4th R&D Institute-4, Agency for Defense Development, Daejeon 305-600, South Korea

**Keywords:** chalcogenides, electronic materials, composites, electrical properties, thermal conductivity

## Abstract

A novel and simple approach was used to disperse Cu nanoparticles uniformly in the Bi_0.5_Sb_1.5_Te_3 _matrix, and the thermoelectric properties were evaluated for the Cu-dispersed Bi_0.5_Sb_1.5_Te_3_. Polycrystalline Bi_0.5_Sb_1.5_Te_3 _powder prepared by encapsulated melting and grinding was dry-mixed with Cu(OAc)_2 _powder. After Cu(OAc)_2 _decomposition, the Cu-dispersed Bi_0.5_Sb_1.5_Te_3 _was hot-pressed. Cu nanoparticles were well-dispersed in the Bi_0.5_Sb_1.5_Te_3 _matrix and acted as effective phonon scattering centers. The electrical conductivity increased systematically with increasing level of Cu nanoparticle dispersion. All specimens had a positive Seebeck coefficient, which confirmed that the electrical charge was transported mainly by holes. The thermoelectric figure of merit was enhanced remarkably over a wide temperature range of 323-523 K.

**PACS**: 72.15.Jf: 72.20.Pa

## Introduction

Thermoelectric materials require a high Seebeck coefficient (*α*), high electrical conductivity (*σ*), and low thermal conductivity (*κ*) at an application temperature (*T *in kelvin) for a high figure of merit (*ZT *= *α^2^σTκ*^-1^), which is related to the thermoelectric energy conversion efficiency. On the other hand, these parameters are not independent for a given material. The quantity, *α^2^σ*, is called the power factor, and the Seebeck coefficient and electrical conductivity are related to the carrier concentration and mobility (effective mass of carriers). The thermal conductivity has contributions from lattice vibrations related to phonon scattering and charge carrier transportations affected by the carrier concentration. The phonon glass and electron crystal (PGEC) concept is considered to reduce the thermal conductivity while maintaining the high power factor by nanostructure engineering [1]. The figure of merit can be enhanced if the nanoparticles are well-dispersed and sufficiently small to intensify phonon scattering without increasing charge carrier scattering [2,3].

In general, the decrease in thermal conductivity by phonon scattering accompanies the electrical conductivity reduction by charge carrier scattering due to the inhomogeneous distribution and agglomeration of nanoparticles [4-6]. A conventional mixing process such as ball milling cannot provide an appropriate dispersion to realize the PGEC effect effectively in composites. In this study, a novel and simple approach was used to prepare the Cu-dispersed Bi_0.5_Sb_1.5_Te_3 _(BAT) composites, and the thermoelectric and transport properties were examined.

## Experimental procedure

A BAT ingot was prepared by melting at 1,073 K for 4 h with high purity (99.999%) Bi, Sb, and Te granules in an evacuated quartz ampoule. The ingot was crushed into powder and sieved to obtain < 75-μm-diameter particles. The Bi_0.5_Sb_1.5_Te_3 _powder was dry-mixed with Cu(OAc)_2 _powder. The resulting Bi_0.5_Sb_1.5_Te_3 _and Cu(OAc)_2 _mixture was transferred to an alumina crucible and heated at 573 K for 3 h in a vacuum to decompose the Cu(OAc)_2 _to Cu nanoparticles, which were bonded chemically to the Bi_0.5_Sb_1.5_Te_3 _powder. Cu-dispersed Bi_0.5_Sb_1.5_Te_3 _composites were hot-pressed in a cylindrical graphite die with an internal diameter of 10 mm at 673 K under a pressure of 70 MPa for 1 h in a vacuum. Scanning electron microscopy (SEM; FEI Quanta400) was used to observe the microstructure. Phase analysis was performed by X-ray diffraction (XRD; Bruker D8 Advance) using Cu Kα radiation. Hall effect measurements were carried out in a constant magnetic field (1 T) and electric current (50 mA) using a Keithley 7065 system at room temperature to determine the carrier concentration and mobility. The Seebeck coefficient and electrical conductivity were measured using temperature differential and four-probe methods, respectively, with Ulvac-Riko ZEM3 equipment in a helium atmosphere. The thermal conductivity was estimated from the thermal diffusivity, specific heat, and density measurements using a laser flash Ulvac-Riko TC9000H system in a vacuum. The thermoelectric figure of merit was evaluated.

## Results and discussion

**Figure 1:**
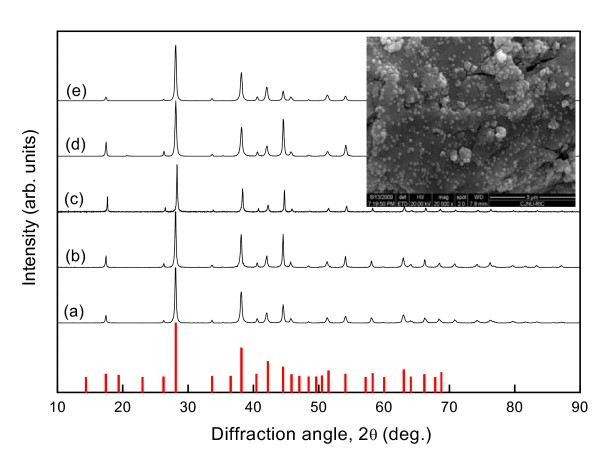
**XRD patterns of Cu-dispersed Bi_0.5_Sb_1.5_Te_3_**. (a) Bi_0.5_Sb_1.5_Te_3 _(BAT), (b) BAT + 0.05 wt.% Cu(OAc)_2_, (c) BAT + 0.1 wt.% Cu(OAc)_2_, (d) BAT + 0.3 wt.% Cu(OAc)_2_, and (e) BAT + 0.5 wt.% Cu(OAc)_2_. The inset is a SEM image of Cu-dispersed Bi_0.5_Sb_1.5_Te_3 _prepared by Cu(OAc)_2 _decomposition.

Figure 1 shows XRD patterns of the Cu-dispersed Bi_0.5_Sb_1.5_Te_3 _prepared by Cu(OAc)_2 _decomposition and then consolidated by hot pressing. The diffraction peaks were well-matched with the International Centre for Diffraction Data standard data. All specimens were polycrystalline with good crystallinity, and the Bi_0.5_Sb_1.5_Te_3 _phase was synthesized successfully using this process. Diffraction peaks for Cu particles were not identified because the amount of Cu was too small to detect. The inset in Figure 1 presents a SEM image of the surface of Cu-dispersed Bi_0.5_Sb_1.5_Te_3 _prepared by heating at 573 K for 3 h in a vacuum to decompose Cu(OAc)_2_. This heat treatment vaporized radical ions of the Cu(OAc)_2 _acetate, resulting in the formation of Cu nanoparticles with a spherical shape. The mean particle size of Cu was approximately 40 nm, which is almost the same as the Cu(OAc)_2 _powder size. Cu nanoparticles were well-dispersed without agglomeration and bonded to the Bi_0.5_Sb_1.5_Te_3 _powder surface.

**Figure 2:**
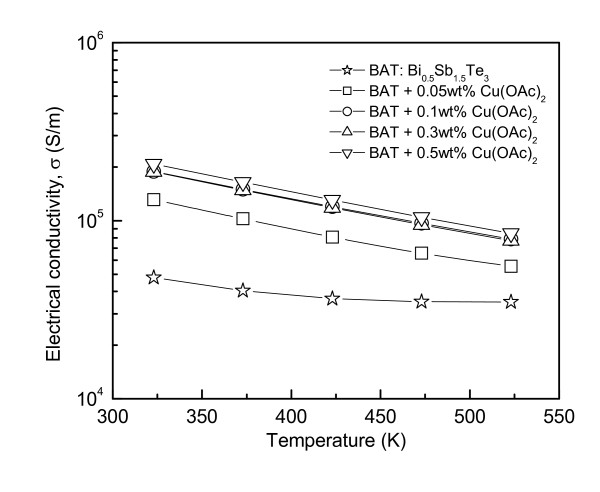
**Electrical conductivity of Cu-dispersed BiBi_0.5_Sb_1.5_Te_3_**.

Figure 2 shows the electrical conductivity of the Cu-dispersed Bi_0.5_Sb_1.5_Te_3_. The electrical conductivity of Bi_0.5_Sb_1.5_Te_3 _was 5 × 10^4 ^S/m at room temperature but increased to 2 × 10^5 ^S/m by Cu dispersion. This increase was attributed to an increase in carrier concentration due to the doping effect from Cu nanoparticles. The electrical conductivity increased systematically with increasing level of Cu nanoparticle dispersion but decreased with increasing temperature similar to that observed with metals or degenerate semiconductors. The relationship between the increase in carrier concentration (*n*) by excitation over the bandgap and the electrical conductivity (*σ*) can be expressed as follows [7]:

(1) $$\sigma =\frac{\text{n}{\text{e}}^{2}\tau }{m^{*}}=\text{ne}\mu$$

where *e *is the electronic charge of the carrier, *τ *is the relaxation time of the carrier, m* is the effective mass of the carrier, and *μ *is the carrier mobility.

The Hall coefficient (*R*_H_), carrier concentration, and mobility were measured to examine the electronic transport properties. Table 1 lists the electronic transport properties of Cu-dispersed Bi_0.5_Sb_1.5_Te_3 _at room temperature. The sign of the Hall coefficient was positive for all specimens, which means that the electrical charge was transported mainly by holes. The carrier concentration of Bi_0.5_Sb_1.5_Te_3 _was 2.4 × 10^19 ^cm^-3 ^but increased to 1.3 × 10^20 ^cm^-3 ^by Cu dispersion. The carrier mobility did not change significantly with Cu dispersion, which indicates that the Cu nanoparticles are too small to introduce charge carrier scattering. Therefore, the electrical conductivity was increased by the Cu dispersion, as shown in Figure 2.

**Table 1:** Electronic transport properties of Cu-dispersed Bi_0.5_Sb_1.5_Te_3 _at room temperature

Specimen	*R*_H _(cm^3^/C)	*n *(cm^-3^)	*μ *(cm^2^/Vs)	m* (*m*_o_)
BAT	0.254	2.4 × 10^19^	121.4	0.85
BAT + 0.05 wt% Cu(OAc)_2_	0.010	6.2 × 10^19^	131.1	1.98
BAT + 0.1 wt% Cu(OAc)_2_	0.046	1.3 × 10^20^	91.5	1.57
BAT + 0.3 wt% Cu(OAc)_2_	0.052	1.2 × 10^20^	97.3	1.47
BAT + 0.5 wt% Cu(OAc)_2_	0.056	1.1 × 10^20^	104.6	1.29

**Figure 3:**
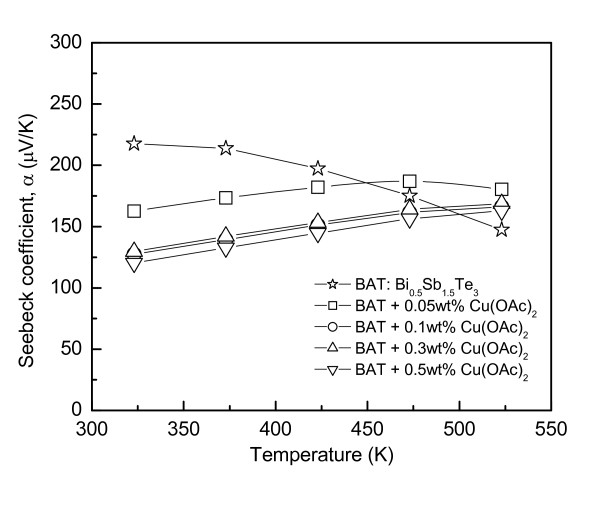
**Seebeck coefficient of Cu-dispersed Bi_0.5_Sb_1.5_Te_3_**.

Figure 3 presents the Seebeck coefficient of Cu-dispersed Bi_0.5_Sb_1.5_Te_3_. All specimens had a positive Seebeck coefficient, which confirmed that the electrical charge was transported mainly by holes, as shown in Table 1. The Seebeck coefficient of Bi_0.5_Sb_1.5_Te_3 _decreased with increasing temperature. It was decreased at room temperature by the Cu dispersion and increased with increasing temperature. The Seebeck coefficient (*α*) of a p-type semiconductor can be expressed as Equation 2 [8,9]:

(2) $$\alpha =\frac{k}{e}\left(\frac{5}{2}+r+\ln\frac{N_{\text{V}}}{n}\right)$$

where *k *is the Boltzmann constant, *r *is the exponent of the power function in the energy-dependent relaxation time expression, and *N*_V _is the effective density of states in the valence band. Therefore, as shown in Figure 3, the Seebeck coefficient of Bi_0.5_Sb_1.5_Te_3 _decreased with increasing temperature due to an increase in carrier concentration by intrinsic conduction. The sign of the Seebeck coefficient was positive, which is in good agreement with the sign of the Hall coefficient, indicating that Bi_0.5_Sb_1.5_Te_3 _is a p-type semiconductor.

The Seebeck coefficient is affected by the carrier concentration and the effective mass and can be expressed by assuming degenerate parabolic band semiconductor properties [10]:

(3) $$\alpha =\frac{8\pi^{2}k^{2}}{3eh^{2}}{\left(\frac{\pi }{3n}\right)}^{2∕3}m^{*}T$$

In this study, the decrease in the Seebeck coefficient of the Cu-dispersed Bi_0.5_Sb_1.5_Te_3 _at room temperature was due to the increase in the carrier concentration. On the other hand, the increase in the Seebeck coefficient of Cu-dispersed Bi_0.5_Sb_1.5_Te_3 _at high temperatures was due to an increase in the effective carrier mass, which is one of the critical factors for determining the Seebeck coefficient. Table 1 lists the change in the effective mass by the Cu dispersion. The charge-carrier energy filtering effect of the nanoparticles was suggested to be the cause of the increase in effective mass [11].

**Figure 4:**
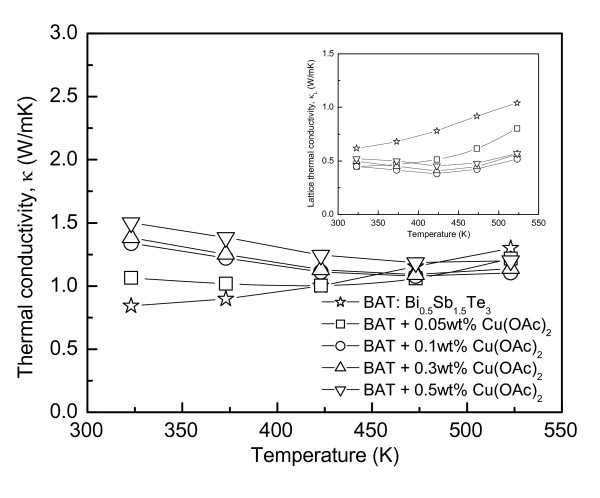
**Thermal conductivity of Cu-dispersed Bi_0.5_Sb_1.5_Te_3_: The inset is the lattice thermal conductivity**.

Figure 4 shows the thermal conductivity of Cu-dispersed Bi_0.5_Sb_1.5_Te_3_. The thermal conductivity of Bi_0.5_Sb_1.5_Te_3 _increased with increasing temperature, whereas that of Cu-dispersed Bi_0.5_Sb_1.5_Te_3 _decreased slightly with increasing temperature. The thermal conductivity increased at room temperature but decreased at higher temperatures as a result of Cu dispersion. The thermal conductivity (*κ*) is the sum of the lattice thermal conductivity (*κ*_L_) by phonons and the electronic thermal conductivity (*κ*_E_) by carriers, and it is given by Equation 4:

(4) $$\kappa =\kappa_{\text{L}}+\kappa_{\text{E}}=\kappa_{\text{L}}+L\sigma T$$

Both components can be separated by the Wiedemann-Franz law (*κ*_E _*= LσT*), where the Lorenz number is assumed to be a constant (*L *= 2.0 × 10^-8 ^V^2 ^K^-2^) for the evaluation [12,13].

The lattice thermal conductivity reduction was expected by the enhancement of phonon scattering at a large density of incoherent interfaces, which was created between the Bi_0.5_Sb_1.5_Te_3 _matrix and Cu nanoparticles. As shown in the inset in Figure 4, the well-controlled incoherent interfaces could behave as effective phonon scattering centers, whereas several reports suggested that coherent interfaces are essential for realizing the PGEC effect effectively [2,3]. The decrease in the lattice thermal conductivity by Cu dispersion increased significantly with increasing temperature. This was attributed to the successful role of Cu nanoparticles as phonon scattering centers. Although the electronic thermal conductivity was increased by Cu nanoparticles due to the increase in carrier concentration, the decrease in the lattice thermal conductivity overcame the electronic thermal conductivity at high temperatures. Therefore, the thermal conductivity was reduced by Cu dispersion at high temperatures, as shown in Figure 4.

**Figure 5:**
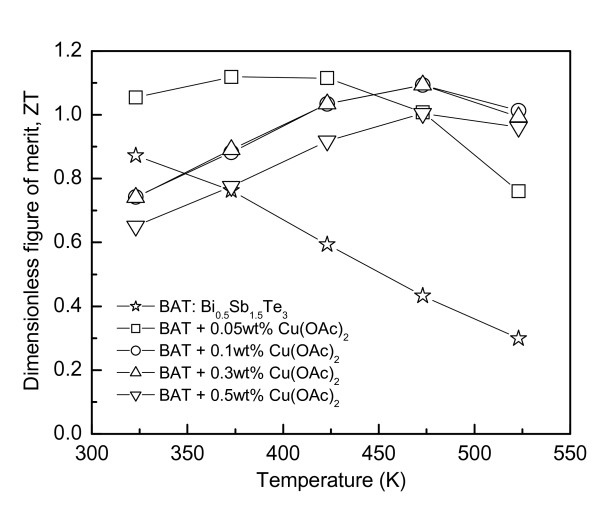
**Thermoelectric figure of merit of Cu-dispersed Bi_0.5_Sb_1.5_Te_3_**.

Figure 5 shows the dimensionless thermoelectric figure of merit (*ZT*) for Cu-dispersed Bi_0.5_Sb_1.5_Te_3_, which was determined by Equation 5 [14]:

(5) $$ZT=\frac{\alpha^{2}\sigma T}{\kappa }\sim {\left(\frac{m^{*}}{m}\right)}^{3∕2}\frac{\mu T^{5∕2}}{\kappa_{\text{L}}}$$

where *m *is the mass of a carrier. Therefore, a superior thermoelectric material should have a large Seebeck coefficient (large effective mass of a carrier), high electrical conductivity (low carrier scattering), and low thermal conductivity (high phonon scattering). The *ZT *value was enhanced dramatically by the Cu nanoparticle dispersion, which was attributed mainly to the increase in power factor. The maximum *ZT *of 1.1 was obtained at 373-423 K for the 0.05 wt.% Cu(OAc)_2 _added Bi_0.5_Sb_1.5_Te_3 _nanocomposite. Compared to Bi_0.5_Sb_1.5_Te_3_, the *ZT *value was improved remarkably by the Cu dispersion, particularly at high temperatures.

## Conclusions

Cu-dispersed Bi_0.5_Sb_1.5_Te_3 _was successfully prepared by Cu(OAc)_2 _decomposition and hot pressing. The Cu nanoparticles were well-dispersed in the Bi_0.5_Sb_1.5_Te_3 _matrix and acted as phonon scattering centers effectively. The electrical conductivity increased systematically with increasing amount of Cu nanoparticle dispersion. The Seebeck coefficient of Bi_0.5_Sb_1.5_Te_3 _decreased with increasing temperature, but its temperature dependence was changed by Cu dispersion. The decrease in lattice thermal conductivity by Cu dispersion overcame the increase in electronic thermal conductivity. The thermoelectric figure of merit was enhanced remarkably over a wide temperature range of 323-523 K due to the high electrical conductivity and the maintenance of low thermal conductivity.

## Competing interests

The authors declare that they have no competing interests.

## Authors' contributions

IHK synthesized the thermoelectric materials. SMC measured the thermoelectric properties. WSS analyzed the transport properties. DIC prepared the composites by acetate decomposition.
